# Is human cytomegalovirus a target in cancer therapy?

**DOI:** 10.18632/oncotarget.383

**Published:** 2011-12-31

**Authors:** John Inge Johnsen, Ninib Baryawno, Cecilia Söderberg-Nauclér

**Affiliations:** ^1^ Childhood Cancer Research Unit, Department of Women's and Children's Health, Karolinska Institute, 171 76 Stockholm, Sweden; ^2^ Karolinska Institute, Department of Medicine, Center for Molecular Medicine, Karolinska University Hospital in Solna, 171 76 Stockholm, Sweden

**Keywords:** cancer, human cytomegalovirus

## Abstract

Human cytomegalovirus (HCMV) is a herpesvirus that is prevalent in the human population. HCMV has recently been implicated in different cancer forms where it may provide mechanisms for oncogenic transformation, oncomodulation and tumour cell immune evasion. Moreover, antiviral treatment against HCMV has been shown to inhibit tumour growth in preclinical models. Here we describe the possible involvement of HCMV in cancer and discuss the potential molecular impact expression of HCMV proteins have on tumour cells and the surrounding tumour microenvironment.

## INTRODUCTION

The interplay between cancer cells and the surrounding microenvironment is essential for the growth and spread of a tumour. The development of malignant tumours requires a microenvironment that supports the uncontrolled proliferation and spread of cancer cells but also conditions that avoid destruction from the various arms of the immune system must be present. The immune system represents an important tool for the destruction of the majority of cancer cells and precancerous conditions in the human body. However, malignant growing tumours have in most, if not all, cases developed immune evasion strategies to avoid destruction by immune cells. One essential immune evasion strategy that can be induced or applied by tumour cells is the formation of an inflammatory microenvironment. Tumour cells can induce inflammation directly through oncogenes that induce transcriptional programs responsible for the production of pro-inflammatory eicosanoids, cytokines and chemokines that attract different cells of the immune system to the microenvironment. Also chronic inflammation caused by viral or microbial infections, autoimmune diseases, dietary products or inflammatory conditions caused by unknown reasons can create an inflammatory microenvironment that support tumour growth [1]. Immune cells that are recruited to the tumour are generally disabled to eliminate tumour cells. Indeed, tumour-related inflammation is regarded as one enabling characteristic crucial for the tumour cell to sustain a proliferative state, evade apoptosis, increase angiogenesis, invasion, metastasis and suppression of immune responses [2].

Although it has been both experimentally difficult and heavily debated, it is today well accepted that approximately 20% of the global cancer burden can be linked to infectious agents including viruses, bacteria and parasites [3]. Recent studies indicate that the list of infectious agents linked to certain cancer forms will increase in the future.

Human cytomegalovirus (HCMV) is a beta-herpesvirus that is common in the human population. Although HCMV is not currently causally implicated in human cancer, a number of recent evidence suggests that HCMV may be specifically associated with some human malignancies. HCMV nucleic acids and proteins have been detected in 90-100% of glioblastomas and medulloblastomas, prostate, breast and colon cancers and in mucoepidermoid carcinomas of salivary glands [4-12]. Consistently, HCMV proteins are not detected in healthy tissues surrounding HCMV positive tumors. HCMV protein expression is restricted to the tumour; mainly in tumour cells, but virus proteins are sometimes found in endothelial cells and inflammatory cells within the tumour. However, infectious virus is not recovered from primary tumours. There is also a discrepancy between the number of protein positive cells and DNA positive cells within the tumour.

We have consistently observed that HCMV proteins are widespread and easily detected in a majority of tumour samples, whereas viral DNA is detected only in few cells within the tumour ([12] and unpublished observations). Recently, Ranganathan et. al. sequenced viral DNA from 20 different HCMV gene regions in samples obtained from glioblastoma patients and also found that only a minority of the cells in the tumour harbour the virus genome [13]. The authors suggested that HCMV may enhance the growth or survival of a tumour through mechanisms that are distinctly different compared to classic tumour viruses that express transforming viral oncoproteins in the tumour cells. Thus, it is not likely that HCMV is an opportunistic virus capable of reactivating in the tumour and then only infects cells within in the tumour. Instead, HCMV proteins, rather than a productive infection may aid the development of HCMV positive tumours through yet undiscovered mechanisms.

## HCMV; A PROMOTER OF CELLULAR TRANSFORMATION OR AN ONCOGENIC VIRUS?

As of today, HCMV is not considered to have direct oncogenic properties; its potential role in cancer seems to be oncomodulatory, which imply that expression of HCMV gene products in cancer cells may promote tumour growth by enabling different hallmarks of cancer [2, 14, 15]. However, numerous recent data also indicate that several HCMV encoded proteins have biological properties that are directly related to cellular transformation and tumour development.

The US28 chemokine receptor encoded by HCMV has several characteristics resembling a viral oncoprotein [16-19]. Expression of US28 in NIH3T3 cells render these cells tumourigenic when injected into nude mice and transgenic mice with targeted expression of US28 to intestinal epithelial cells results in the development of intestinal neoplasia, which can be enhanced by inflammation [16]. US28 targeted expression in intestinal cells inhibits glycogen synthase-3β (GSK-3β) function resulting in increased β-catenin activity and induced expression of Wnt target genes, including *cyclin D*, *survivin* and *c-myc*, that are involved in the control of cell proliferation [16]. These findings provide a direct molecular link between the expression of US28 and oncogenesis. In addition, US28 has also been shown to activate the transcription factor nuclear factor κB (NF-κb) that is a critical regulator of immunity, stress responses, apoptosis and differentiation [19, 20].

In glioblastoma cells, we found that the HCMV IE72 protein directly interacts with the hTERT promoter at SP1 binding sites to induce telomerase activity and telomere lengthening [4]. We also found that HCMV-IE72 and hTERT were co-expressed in primary glioblastoma samples [4]. Enhanced telomerase activity is necessary for tumour cells to divide indefinitely and is commonly induced by oncogenic viruses [21]. Recently, Melnick et al. suggested that HCMV fulfils the criteria of Koch's Postulates as revised for viruses and cancer, and that HCMV therefore should be designated as an “oncovirus” [9]. They demonstrated cell specific localization of HCMV in 97% of mucoepidermoid carcinomas of salivary glands. HCMV IE and pp65 were expressed in tumour cells, but not in non-tumour cells and positively correlated with severity. HCMV protein expression correlated with activation of known oncogenic pathways such as epidermal growth factor receptor (EGFR), cyclooxygenase-2 (COX-2), Erk and amphiregulin. They also used a mouse salivary gland organ culture model and showed that murine CMV infection induces dysplasia through an upregulation of Erk phosphorylation. Phosphorylation of the ErbB receptor family members and downstream signalling may therefore be relevant targets for drug discovery also of HCMV positive tumours [9, 22].

The interaction of HCMV with its cellular receptor ligands, like integrins, during infection results in the activation of the PI3K/Akt signalling pathway and expression of IE72 protein in glioblastoma cells induces constitutive activation of Akt [23, 24]. HCMV has been shown to also activate the PI3K/Akt signalling cascade via binding of HCMV proteins to platelet-derived growth factor receptor alpha (PDGFR) and by selective phosphorylation of the cellular focal adhesion kinase (FAK) in glioblastoma and prostate cancer cells [25-27]. Furthermore, HCMV UL38 was shown to interact with tuberous sclerosis complex resulting in dysregulation of the mammalian target of rapamycin complex 1 [28].

HCMV encodes several proteins that interfere with the cellular apoptotic machinery. Direct anti-apoptotic activity of HCMV proteins has been located to transcripts encoded by the HCMV UL36-UL38 genes [29]. CMV blocks apoptosis mediated by death receptors and encodes a mitochondria-localized inhibitor of apoptosis that suppresses apoptosis induced by diverse stimuli. The HCMV UL37 gene product inhibits Fas-mediated apoptosis downstream of caspase-8 activation and Bid cleavage in the mitochondria through inhibition of the pro-apoptotic Bcl-2 family members Bax and Bak [30, 31]. The HCMV UL36 gene product inhibits Fas-mediated apoptosis by binding to and inhibiting the function of caspase-8. [32]. HCMV infection has also been shown to inhibit apoptosis and induce drug resistance by induction of the p53 tumour suppressor homologue gene product ΔN-p73α, resulting in abnormal neural cell survival [33]. The HCMV IE86 protein binds to p53 and inhibits its transactivating function and suppresses p53-mediated apoptosis after DNA damage [26, 34-37]. The HCMV UL97 protein is a viral homologue of cellular cyclin-dependent kinases (CDK) that phosphorylates and inactivates the retinoblastoma (Rb) tumour suppressor protein resulting in cell cycle progression and inhibition of apoptosis in mammalian cells [38].

The functional inhibition of the p53 and Rb families of tumour suppressor proteins by HCMV encoded proteins implicates that HCMV is able to promote cell cycle progression, increase DNA synthesis and block apoptosis resulting in increased chromosomal instability [39-43]. In neuroblastoma cells HCMV induces expression of Bcl-2 resulting in inhibition of apoptosis and chemoresistance, a process that can be reversed by treatment of neuroblastoma cells with the antiviral drug ganciclovir [44]. Interestingly, case reports of neuroblastoma patients have shown increased HCMV antibody titers and detection of HCMV in urine of small children with neuroblastoma[45]. HCMV DNA also has been detected in neuroblastoma tissue sample [45-47]. Unpublished results from our laboratory demonstrate HCMV DNA, RNA and proteins in the majority of neuroblastoma tissue samples and in neuroblastoma cell lines. Treatment of neuroblastoma cells with the anti-viral drug ganciclovir *in vitro* or *in vivo* inhibits tumour growth (Wolmer-Solberg 2011, submitted).

Hence, HCMV encodes for a number of different proteins that have profound effects on cellular processes leading to increased proliferation, inhibition of apoptosis, stimulation of cellular migration, the release stimulatory factors, induction chemotherapeutic resistance and increased telomerase activity.

## HUMAN CYTOMEGALOVIRUS; AN ENHANCER OF INFLAMMATION AND INDUCER OF IMMUNE EVASION IN THE TUMOUR MICROENVIRONMENT

Symptoms of a primary HCMV infection are usually mild or asymptomatic in immunocompetent individuals but can cause severe disease in fetuses and immunocompromised patients such as transplant recipients and AIDS patients. The virus is spread through all bodily fluids and establishes a life-long latent/persistent infection. Reactivation from latency appears to be triggered by inflammation, which the virus can initiate by inducing cytokine and chemokine production and by enhancing the synthesis of pro-inflammatory eicosanoids. Indeed, the biological responses elicited by HCMV reactivation mimic those seen in leukocyte dysfunction, wound healing and chronic inflammation [14]. HCMV reactivation has also been shown to stimulate the expression of VEGF that can induce angiogenesis [17, 18] and inhibit the expression of the potent anti-angiogenic protein thrombospondin-1 [48].

During evolution HCMV has coevolved with the human host and the virus has developed several immune evasion strategies to allow persistent infection and viral spread without harming its host. HCMV contains a 250 kb ds DNA genome that has 252 open reading frames and encodes approximately 200 proteins, of which only about 50 are essential for viral replication [49]. Hence, the majority of HCMV encoded proteins have other functions in the viral lifecycle and many of these proteins are involved in immune evasion. For instance, the US11, US2 and US3 gene products prevent host cell MHC class I antigen expression that is required for CD8+ cytotoxic tumour killing. HCMV also induces a specific block in presentation of peptides of the HCMV encoded IE1 protein; one of the earliest immunodominant HCMV epitopes [50-52]. US3 and US8 inhibit presentation of MHC class II molecules on the cell surface and thereby inhibit CD4 + T cell responses [53, 54]. The HCMV pp65 protein encoded by the UL83 gene redirect HLA class II molecules to lysosomes where the alpha chain of the HLADR molecule is degraded [55]. HCMV inhibits NK mediated lysis by several different strategies; the virus encodes for an MHC class I homologue that prevents NK cells to become activated through the missing self-hypothesis. The viral protein UL16 retains the NKG2D ligands ULBP1, 2 and MIC-B in the ER that are essential to activate an NK cell response (reviewed in [56]). UL16 also protects the cells from lysis mediated by cytotoxic peptides [57]. Thus, cancer cells expressing UL16 would be protected against the action of both NK cells and T cells. Interestingly, the HCMV encoded UL83 protein pp65 and IE1/IE2 are frequently detected in both gliomas and medulloblastomas [11, 12].

We recently showed that HCMV nucleic acids and proteins are present in the majority of medulloblastoma primary tumours and cell lines. We also found that US28 (the HCMV encoded chemokine receptor homologue with potential oncogenic functions) was expressed in medulloblastoma and induced expression of COX-2 in these tumours [12]. Microarray analysis of US28 transfected cells and HCMV infected cells showed that the expression of COX-2 is highly up-regulated in these cells as compared to mock-transfected or HCMV negative cells [17, 39]. Moreover, transgenic mice with targeted expression of US28 to intestinal epithelial cells exhibit a hyperplastic intestinal epithelium resulting in tumour development, indicating that US28 is involved in tumour initiation and progression [16].

COX-2 is over- expressed in a number of different adult cancers of epithelial origin as well as in gliomas where high expression often is correlated with poor prognosis (reviewed in [58-60]). In paediatric solid tumours high expression of COX-2 has been found in neuroblastoma [61, 62], medulloblastoma [63, 64] and sarcomas [65]. COX-2 is one of the major enzymes responsible for the conversion of arachidonic acid to the pro-inflammatory eicosanoid, prostaglandin E_2_ (PGE_2_). Increased levels of prostaglandin E_2_ (PGE_2_) are perceived in malignancies of different origin, including brain tumors [66-68]. PGE_2_ exerts its physiological effects by interacting with a subfamily of four distinct G-protein–coupled receptors designated EP_1_, EP_2_, EP_3_, and EP_4_. PGE_2_ promotes tumour growth in an autocrine and/or paracrine manner by stimulating EP receptor signalling with subsequent enhancement of cellular proliferation, promotion of angiogenesis, inhibition of apoptosis and stimulation of invasion [58]. In addition, PGE_2_ is an important mediator for the interaction between tumour cells and cells in the tumor microenvironment where PGE_2_ contributes to the generation of a tumor promoting inflammatory microenvironment that suppress the activities from cells in the immune system [58].

Different nonsteroidal anti-inflammatory drugs (NSAIDs) which inhibit the enzymatic function of cyclooxygenases and the production of prostaglandins and other inflammatory mediators has been shown to be promising agents for the prevention and treatment of various cancers [69]. Elevated levels of PGE_2_ are required for efficient replication of HCMV by facilitating the production of the HCMV immediate-early 2 protein [70]. Daily aspirin reduce both the risk of development of cancer and cancer deaths [71]; the benefit increased with duration of treatment [72]. Interestingly, NSAIDs abrogate virus-mediated production of PGE_2_ and reduce the virus burden in HCMV infected cells [70, 73]; thus acting as an anti-viral agent against HCMV. Moreover, the COX-2 specific NSAID celecoxib reduces the levels of PGE_2_ and the expression of HCMV proteins in medulloblastoma, as well as tumour growth *in vitro* and *in vivo* [12].

US28 that induces the expression of COX-2 in HCMV infected cells can bind different chemokines, including CCL2, CCL5, and CX3CL1 [74], and suppress the host immune responses [75]. Moreover, US28 activates NF- κB resulting in activation of the IL-6–JAK1–STAT3 signalling axis and increased interleukin-6 (IL-6), VEGF and endothelial nitric oxide synthase (e-NOS) production [14, 19]. Analysis of clinical glioblastoma samples *in situ* showed co-localization of US28 with phosphorylated STAT3, COX-2, VEGF and e-NOS, suggesting that US28 in addition to promoting an inflammatory microenvironment also contribute to tumour invasiveness and angiogenesis [14, 19]. Taken together US28 could provide a target for therapy in HCMV-positive tumours.

HCMV establishes latency in myeloid lineage cells, and reactivation is dependent on inflammation and differentiation of monocytes into macrophages of dendritic cells. HCMV can also persistently infect monocyte/macrophage lineage cells and induce a strong inflammatory response in these cells [76]. In human breast and colon cancer HCMV protein expression has been detected in infiltrating inflammatory cells in the tumour microenvironment and in gliomas, macrophages and microglia cells as well as tumor cells exhibit positive HCMV protein staining [77, 78]. HCMV infection of moncyte/macrophages is associated with an induction of IL-1, IL-6, IL-10, TNF-α and TGF-β that are potent cytokines with both immune stimulating and immunosuppressive effects on the host anti-tumour response [1, 79]. In particular, CMVIL-10 and TGF-β would provide an immunosuppressive microenvironment in HCMV positive tumours [80, 81]. These evidences raise the prospect that a persistent HCMV infection could induce the same kind of “smoldering” inflammation at the same time as it creates an immunosuppressive environment, which is frequently observed in the tumour microenvironment [1, 78].

## HCMV AS A GUARDIAN OF CANCER STEM CELLS

HCMV is a neurotropic virus that can persistently infect neural precursor cells. As a consequence HCMV is the major infectious cause of birth defects in infants, including sensori-neural hearing loss or neuronal migration disturbances during brain development, and in the most severe cases, microcephaly or anencephaly. We have demonstrated that HCMV can block the ability of neural progenitor cells to differentiate into neurons or astrocytes [82, 83]. HCMV DNA and gene products have repeatedly been detected by several laboratories in preneoplastic and neoplastic tumour cells in human glioblastoma tissue samples and the fractions of tumour cells infected with HCMV correlate significantly with tumour staging and patient survival [5, 84]. We recently reported that the majority of primary human medulloblastoma and cell lines propagated for years in laboratories contain HCMV DNA, RNA and express HCMV IE and late proteins [12]. Our unpublished data also demonstrate that HCMV is present in the majority of childhood primary neuroblastoma and cell lines, an observation which is consistent with other reports [15, 45].

Medulloblastoma and neuroblastoma are embryonal tumours of the central and peripheral nervous systems, respectively. Compared to adult tumours, paediatric tumours generally have a dramatically shortened latency period and harbour fewer genetic aberrations causing oncogene activation or loss of apoptotic regulators. The reason for these differences is that these malignancies probably arise from stem or progenitor cells which already possess proliferative capacity as a part of the normal developmental process [85]. Medulloblastoma and neuroblastoma are linked to dysfunctional pathways that are operative during normal development [85]. The clinical presentation and treatment response also suggests that a tumour initiating cell population exist in these tumours [86-91].

Although the cellular origin of gliomas still is contended, recent evidence suggests that multipotent neural stem or progenitors of the subventricular zone (SVZ) are cells with the potential to form gliomas [92]. Subpopulations of CD133^+^ and/or CD15^+^ cells in both medulloblastomas and glioblastomas have been recognized as potential cancer stem cells [89, 93]. In neuroblastoma, on the other hand, no true marker for potential cancer stem cells have been found, although CD133 and CD44 are implied as potential markers [88]. We have detected HCMV DNA, RNA and proteins in medulloblastoma, glioblastoma and neuroblastoma cell lines used world-wide for decades in laboratories, which may indicate that the virus in condemned in a stem cell that is maintained in culture and gives rise to tumours [12]. We observed that the expression of HCMV proteins in both medulloblastoma and neuroblastoma cell lines varied considerably between different sampling occasions over a one year period, and that protein expression increased when the cells were engrafted in nude mice. We therefore hypothesize that HCMV DNA and proteins are maintained in a stem-cell like phenotype. Indeed we observed HCMV protein expression in the majority of CD133+ medulloblastoma cells whereas in neuroblastoma this number varied between 4-34% depending on cell line and sampling time ([12], and unpublished observations). Likewise, in glioblastoma tissue samples 40-60% of the CD133^+^ cell population expressed HCMV IE1 [14] and our own unpublished observations). These data indicate that HCMV is present in tumour cells that express stem cell markers, and that the virus is maintained in cell lines over long periods of time. The fact that HCMV is able to inhibit the differentiation of neural progenitor cells raises the possibility that HCMV encoded proteins are involved in the maintenance of a cancer stem cell population within neural tumours.

## ANTI-HCMV THERAPY AS A TREATMENT OPTION FOR CERTAIN CANCERS

The findings that several cancer forms are HCMV positive, including those with a neural origin that usually have a dismal prognosis, opens up the possibility to treat these cancers with anti-viral drugs against HCMV. In nude mice engrafted with human medulloblastoma cells, the antiviral drug valganciclovir, significantly inhibited tumour growth. Interestingly the treatment effect was extensively enhanced when valganciclovir was combined with the COX-2 specific inhibitor celecoxib [12], which is known to also inhibit HCMV infection. Importantly, the inhibition of tumour growth clearly corresponded with reduction in the expression of late HCMV proteins in these tumours. However, neither valganciclovir by itself or in combination with celecoxib was able to completely eliminate the HCMV presence. In sharp contrast, valganciclovir had no effect neither on the clonogenic capacity or tumour growth of two HCMV-negative cell lines derived from prostate and pancreas adenocarcinomas [12]. This strongly suggests that the inhibitory effect of valganciclovir on medulloblastoma growth is HCMV specific and not mediated by potential non-specific drug effects inhibiting cellular proliferation.

Medulloblastoma, neuroblastoma and glioblastoma tumors express high levels of COX-2 and NSAIDs, inhibitors of COX-2 and PGE2 production, have profound effects on the growth of these tumours [64, 94-96]. These inhibitors also efficiently prevent HCMV replication and reduce the growth of US28-expressing tumour cells [17, 18, 70, 73]. Hence, the beneficial effects seen with aspirin and other NSAIDs in cancer prevention studies could partly be due to inhibition of HCMV replication in pre-malignant lesions. Compared to conventional chemotherapeutic drugs currently used for the treatment of these tumours, both antiviral drugs for HCMV and NSAIDs are well tolerated. Hence, these drugs should undergo clinical testing in combination with conventional therapies in patients carrying HCMV-infected tumours.

In a randomized double-blinded phase II study we are currently evaluating antiviral drugs against HCMV as an adjuvant therapy for glioblastoma. Results from this study are expected to be ready soon. Also a phase I/II immunotherapy clinical trial of autologous HCMV pp65 RNA loaded dendritic cells has been initiated in which 13 patients with newly diagnosed glioblastoma multiforme were enrolled. Initial results from this study are promising. Patients exhibited a median progression-free survival of 15.4 months and overall survival of 20.6 months, numbers which are highly significant compared to historical controls [97].

The promising preclinical and clinical results obtained using antiviral drugs against HCMV to treat tumours carrying HCMV should be extended to include larger controlled clinical trials. Also, developing drugs that specifically inhibit the functions of HCMV encoded US28 may be of future benefit in cancer treatment since the US28 protein may possess important functions in tumour initiation through the activation of intracellular signalling pathways, angiogenesis and effects on the tumour microenvironment.

## CONCLUSIONS AND PERSPECTIVES

The presence and functions of HCMV in cancer is still debated and scepticism vestiges regarding the relationship between HCMV and cancer. This mainly originates from conflicting results regarding the detection of HCMV in tumour samples and since HCMV by itself not has been shown to transform normal cells into cancer cells [47]. The last statement has recently been challenged since the HCMV encoded chemokine receptor homologue US28 renders NIH3T3 cells tumorigenic when injected into nude mice and transgenic mice with targeted expression to intestinal epithelial cells develop intestinal neoplasia [16, 18, 19]. Compared to the high degree of HCMV replication and protein expression seen in primary HCMV infections and in HCMV reactivation in immunocompromised individuals, the expression of viral proteins in cancer cells is very low. The term “microinfection” has been used to describe the low levels of HCMV infection found in cancer [84]. Clearly, the infection is different in cells that replicate the virus and produce infectious virus compared to tumour cells in vivo; in spite of the fact that several HCMV proteins are expressed, infectious virus are not isolated from tumour cells of primary tumors, primary tumour cell cultures or established tumour cell lines. Therefore, as detection of HCMV in cancer cells using standard protocols developed for the detection of active HCMV infection associated with a high HCMV replication rate and high-level expression of HCMV proteins is usually insufficient in these cases, it is believed that low levels of HCMV exists in tumours [13, 15]. However, using flow cytometry examining fresh tumour cells or indirect immunofluorescence examining frozen tumor biopsy specimens, we demonstrated the feasibility of detecting HCMV proteins in primary tumour cells from medulloblastoma, glioblastoma and neuroblastoma patients (Wolmer-solberg, submitted, [12, 98]). Research laboratories that have shown a high prevalence of HCMV nucleic acids and proteins in tumour samples have used highly sensitive immunohistochemical and molecular methods in order to detect the presence of HCMV.

As of today HCMV has been detected in glioma, medulloblastoma, neuroblastoma, breast, prostate and colon cancer and mucoepidermoid tumors of the salivary gland. Although the exact molecular functions of HCMV in these tumours still need to be further investigated, the findings that antiviral HCMV treatment inhibit the growth of certain tumours ([12], Wolmer-Solberg, unpublished) is exciting and future studies will elucidate whether these antiviral therapies should be included as an adjuvant treatment for patients having HCMV-positive tumours.
